# Loss of full length CtBP1 expression enhances the invasive potential of human melanoma

**DOI:** 10.1186/1471-2407-9-52

**Published:** 2009-02-12

**Authors:** Andreas Winklmeier, Ina Poser, Keith S Hoek, Anja K Bosserhoff

**Affiliations:** 1 Institute of Pathology, University Regensburg, 93053 Regensburg, Germany; 2 MPI for Mol. Cell Biology and Genetics, Dresden, Germany; 3 Dept of Dermatology, University of Zurich, Zurich, Switzerland

## Abstract

**Background:**

The C-terminal binding protein 1 (CtBP1) is a known co-repressor of gene transcription. We recently revealed that CtBP1 expression is lost in melanoma cells and melanoma inhibitory activity (MIA) expression is subsequently increased. The present study was performed to evaluate a more general role of CtBP1 in human melanoma and identify further CtBP1-regulated target genes.

**Methods:**

Sequence analysis and expression profile of CtBP1 in melanoma cell lines were done by PCR. Boyden Chamber assays and co-immunoprecipitation were performed to investigate the functional role of CtBP1. Gene expression analysis and micro array data were used to define target genes.

**Results:**

Interestingly, we detected an alternative splice product of CtBP1 with unknown function whose expression is induced at reduction of full length CtBP1. Overexpression of full length CtBP1 in melanoma cells had no effect on cell proliferation but did influence cell migration and invasiveness. To understand the effect of CtBP1 we identified putative LEF/TCF target genes found to be strongly expressed in melanoma using DNA microarray analysis. We focused on fourteen genes not previously associated with melanoma. Detailed analysis revealed that most of these were known to be involved in tumor metastasis. Eleven genes had expression profiles associated with melanoma cell invasiveness.

**Conclusion:**

In summary, this study revealed that reduction of CtBP1 expression is correlated with migratory, invasive potential of melanoma cells.

## Background

CtBP1 was first identified as a cytoplasmatic protein which binds the COOH-terminal region of the adenoviral protein E1A to attenuate its ability to activate transcription. CtBP1 recognizes PXDLS and RRT motifs in DNA-binding proteins and functions as a transcriptional co-repressor in *Drosophila, Xenopus*, and mammals. CtBP1 contains a central NAD(H)-binding domain and exhibits a weak dehydrogenase activity. This domain is also important for dimerization of CtBP1 contributing to the recruitment of transcriptional regulators. Recent studies revealed that the repressor function of CtBP1 is mediated by binding to enzymes catalyzing histone modification as class 1 HDACs (for review see [1]). A number of studies have provided evidence that CtBP1 binds to and regulates HMG-box proteins as TCF4 [2] implicating them in context-dependent transcriptional repression. Transcription factors of the LEF/TCF family contain a homologous HMG-box, recognizes a conserved consensus sequence and regulates expression of genes also involved in melanoma development [3]. In previous work we could show that CtBP1 functions as a strong repressor of melanoma inhibitory activity (MIA) expression by negatively regulating *MIA *promoter activity in malignant melanoma, and that this repressor function requires the TCF binding element in the *MIA *promoter [4]. Expression of MIA is an early event in melanoma development and correlates with tumor progression *in vivo*. MIA promotes invasion and metastasis of melanoma cells regulating cell-matrix attachment (for review see [5]).

CtBPs has a wide impact in tumorigenesis interacting with transcriptional repressors influencing oncogenic and pro-survival pathways (for review see [6]). It was previously shown that CtBP interacts with Snail contributing to tumor progression [7,8]. Further analyses revealed CtBP1 to be strongly expressed in primary melanocytes whereas in melanoma cells *in vitro *and *in vivo *full length CtBP1 expression is lost or strongly downregulated suggesting an important role of CtBP1 in melanoma progression [3]. To define the role of CtBP1 in melanoma more clearly we performed a screening for CtBP1 target genes.

## Methods

### Cell culture

The melanoma cell lines Mel Im hi, Mel Im si, Mel Im, Mel Ju, Mel Juso, Mel Ho, Mel Ei, Mel Wei, Sk Mel 3, Sk Mel 28 and HTZ 19d were described previously [9,10]. Cells were maintained in DMEM supplemented with penicillin (400 U/ml), streptomycin (50 μg/ml), L-glutamine (300 μg/ml) and 10% fetal calf serum (FCS; Sigma, Deisenhofen, Germany) and split at a 1:5 ratio every three days. Normal human epidermal melanocytes (NHEM) derived from normal skin were cultivated in melanocyte medium MGM-3 (Promocell, Heidelberg, Germany) under a humidified atmosphere of 5% CO_2 _at 37°C. Cell proliferation was determined using the XTT assay (Roche, Mannheim, Germany).

A panel of Mel Im cell clones with CtBP1 expression was established by stable transfection using a pCMX-PL1-CtBP1 expression plasmid [4]. Plasmids were cotransfected with pcDNA3 (Invitrogen), containing the selectable marker for neomycin resistance. Mock controls received pcDNA3 alone. Transfections were performed using lipofectamin plus (Invitrogen). One day after transfection, cells were placed in selection medium containing 50 μg/ml G418 (Sigma). After 25 days of selection, individual G418-resistant colonies were subcloned.

### RNA isolation and reverse transcription

Total cellular RNA was isolated from cultured cells (4 × 10^6^). cDNAs were generated by reverse transcriptase reaction performed in 20 μl reaction volume containing 2 μg of total cellular RNA, 4 μl of 5× first strand buffer (Invitrogen, Groningen, The Netherlands), 2 μl of 0.1 M DTT, 1 μl of dN_6_-primer (10 mM), 1 μl of dNTPs (10 mM) and DEPC-water. The reaction mixture was incubated for 10 min at 70°C, 200 units of Superscript II reverse transcriptase (Invitrogen) were added and RNAs were reverse transcribed for 1 hour at 37°C. Reverse transcriptase was inactivated at 70°C for 10 minutes and the RNA was degraded by digestion with 1 μl RNase A (10 mg/ml) at 37°C for 30 minutes. The quality of cDNA was controlled and normalized by RT-PCR amplification of the house keeping gene β-actin.

### Analysis of gene expression

Quantitative real time-PCR was performed on a Lightcycler (TaKaRa, Bio, USA). cDNA template (2 μl), 0.5 μl (20 mM) of forward and reverse primers (Table 1) and 10 μl of SYBR Premix *Ex Taq *in a total of 20 μl were applied to the following PCR program: 10 min 95°C (initial denaturation); 20°C/sec temperature transition rate up to 95°C for 15 sec, 3 sec 64°C, 5 sec 72°C, 86°C acquisition mode single, repeated for 40 times (amplification). Each analysis was performed at least in triplicate. The PCR reaction was evaluated by melting curve analysis according to the manufacturer's instruction and checking the PCR products on 1.8% agarose gels. The quality of cDNA was controlled and normalized by RT-PCR amplification of the house keeping gene β-actin.

**Table 1:** Primers used for quantitative RT-PCR

Gene name	for	rev
AP1S1	GGAGGAGATGGGTTTGGCAT	GTGGAGGGAGGGAATGTTTGA
ATP1B1	TGGCTGGCATCTTCATCGGA	CTTTCGGTTCACTGGGCACA
CLDN11	TCTGTTGCTCAGGCTGGAGT	CGAGGCGGGAGGATACTTTGA
COL1A2	CCCAGCCGGAGATAGAGG	TCACCAGGCTCACCAGCAGG
CtBP1	CGACCTCCGATCATGAAC	GCTAAAGCTGAAGGGTTCC
ENC1	GCAACTTCCAAACCATCAGGA	TCTGGGAGGTAGCAATAGCG
ENPP2	GGAGAGTCGCATTGGGTTGA	TGTAGGGAGAGTGTGTGCCA
FAT	CCAATGATAATCCACCCGAGTT	TAACAACACCCGTCACGC
FSCN1	CCTGGGCGTGTAGTGTAACT	CACCACAAGGGTCAGTCCTA
FUS	TTCGTTGCTTGCTTGCCTG	TGTAACTCTGCTGTCCGTAGG
MLL5	TGGGCTTGTATCTGGTTTCGG	CTGGTGTTGGTAAAGGTAGGCTA
SLC26A2	GATTGGTGAGACAGTTGACCG	TTGAAAGAAGCCCATCGCTAC
THBS1	ACTGCGTTGGTGATGTAACAG	GTGCTCTCCATTGTGGTTGAA
TMED4	TGGGATAAGCAGAAGGAGGTC	ATCTCAGGGTAGTTGTTGGCA
VCAN	TCAGAACAGCAAGTGGCAGCGA	CAACACAAGTGGCTCCATTACGAC

For further analysis of gene expression, we used the micro array data of Smith *et al. *(GDS1989 [11]) and Hoek *et al. *[12]. Via Pigment Cell & Melanoma Research Homepage http://www.pigment.org/genearray_home.asp, these data are available for public.

Sequence analysis of the splice donor and splice acceptor site of CtBP exon 4 was performed on a DNA fragment amplified by conventional PCR using the primers CtBP1 Exon4 for: 5'-TTC AAA CCC GCT CCA GTC-3' and CtBP1 Exon4 rev: 5'-TCA GTC CTG TCC TGG GT TG-3'.

### Western Blot analysis

3 × 10^6 ^cells were lysed in 200 μl RIPA-buffer (Roche) and incubated for 15 minutes at 4°C. Insoluble fragments were removed by centrifugation at 13000 rpm for 10 minutes and the supernatant lysate was immediately shock frozen and stored at -80°C. 40 μg RIPA-cell lysate of each sample of melanoma cell lines were loaded and separated on 8.75% SDS-PAGE gels and subsequently blotted onto a PVDF membrane (1 hour, 150 mA). After blocking for 1 hour with 3% BSA/PBS the membrane was incubated for 16 hours with the primary antibody (anti-CtBP1 antibody, Transduction Lab, 1:2500) or beta-actin (Sigma, 1:2500). Then the membrane was washed three times in PBS, incubated for 1 hour with 1:4000 of an alkaline phosphate-coupled secondary antibody (Chemicon) and then washed again. Finally immunoreactions were visualized by NBT/BCIP (Sigma) staining.

### Co-Immunoprecipitation (Co-IP)

For co-immunoprecipitation cell lysates dissolved in binding buffer (20 mM NaPO_4_, 150 mM NaCl, pH 7.5) were incubated with 2 μg of the anti-TCF4 [4] or anti-Snail antibody (Cell Signaling, USA) with shaking at 4°C overnight. Then 20 μl protein G Sepharose 4 Fast Flow (Amersham, Biosciences) was added for 1 h, pelleted, washed three times with binding buffer, resuspended in 20 μl of Laemmli's buffer, heated at 95°C for 5 min, separated on 10% SDS-polyacrylamide gels and subsequently blotted onto a PVDF membrane (1 hour, 150 mA). After blocking for 1 hour with 3% BSA/PBS the membrane was incubated for 16 hours with the primary antibody (anti-CtBP1 antibody (1:2500)). Then the membrane was washed three times in PBS, incubated for 1 hour with 1:2000 of an alkaline phosphate-coupled secondary antibody (Chemicon) and then washed again. Finally, immunoreactions were visualized by NBT/BCIP (Sigma) staining.

### Migration and invasion assay

Migration and invasion assays were performed using Boyden Chambers containing polycarbonate filters with 8 μm pore size (Costar, Bodenheim, Germany), essentially as described previously [13]. Filters were coated with gelatine or Matrigel (diluted 1:3 in H_2_O; Becton Dickinson, Heidelberg, Germany), respectively. The lower compartment was filled with fibroblast-conditioned medium, used as a chemo-attractant. Mel Im cells and CtBP1 cell clones were harvested by trypsinization for 2 min, resuspended in DMEM without FCS at a density of 3 × 10^4 ^cells/ml (migration) and 2 × 10^5 ^cells/ml (invasion) and placed in the upper compartment of the chamber. After incubation at 37°C for 4 hours, the filters were collected and the cells adhering to the lower surface fixed, stained and counted. Each assay was repeated at least three times.

### Statistical analysis

Results are expressed as mean ± SD (range) or percent. Comparison between groups was made using the Student's unpaired t-test. A p value < 0.05 was considered statistically significant. All calculations were performed using the GraphPad Prism software (GraphPad software Inc, San Diego, USA).

## Results

**Figure 1:**
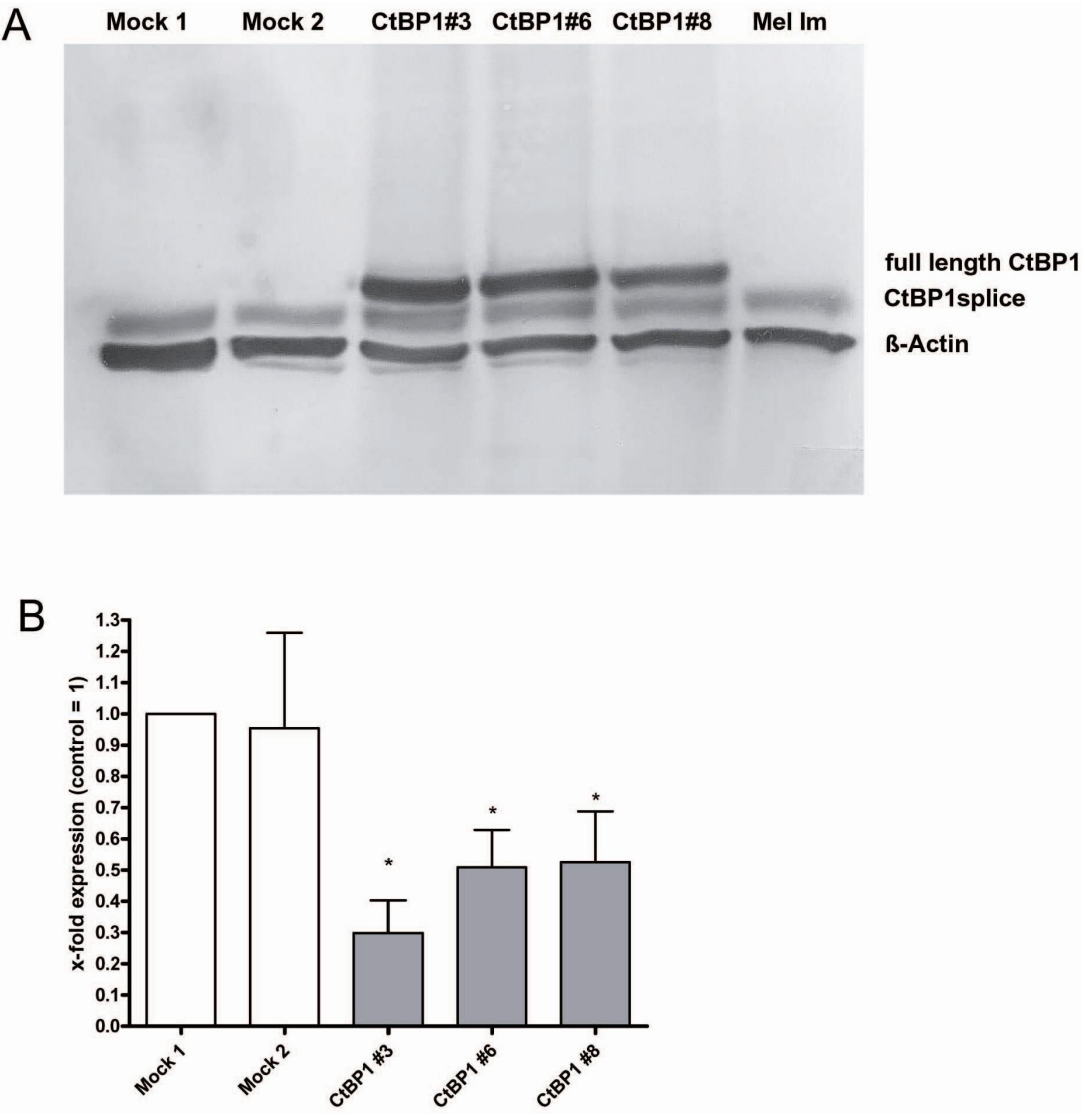
**CtBP1 re-expressing melanoma cell clones**. An expression plasmid for CtBP1 was introduced into Mel Im cells by stable transfection. (**A**) Expression of CtBP1 protein was analyzed by western blotting. Expression of full length CtBP1 was observed in the CtBP1-transfected cell clone CtBP1#3, CtBP1#6 and CtBP1#8 but not in the wild-type Mel Im cell line or in the mock transfected cell clones Mock 1 and Mock 2. (**B**) Influence of CtBP1 expression on MIA mRNA expression was determined by quantitative RT-PCR. The three CtBP1 re-expressing clones (CtBP1#3, #6 and #8) showed strong reduction in MIA expression compared with mock transfected clones. (*: p < 0.05 compared to Mock 1).

**Figure 2:**
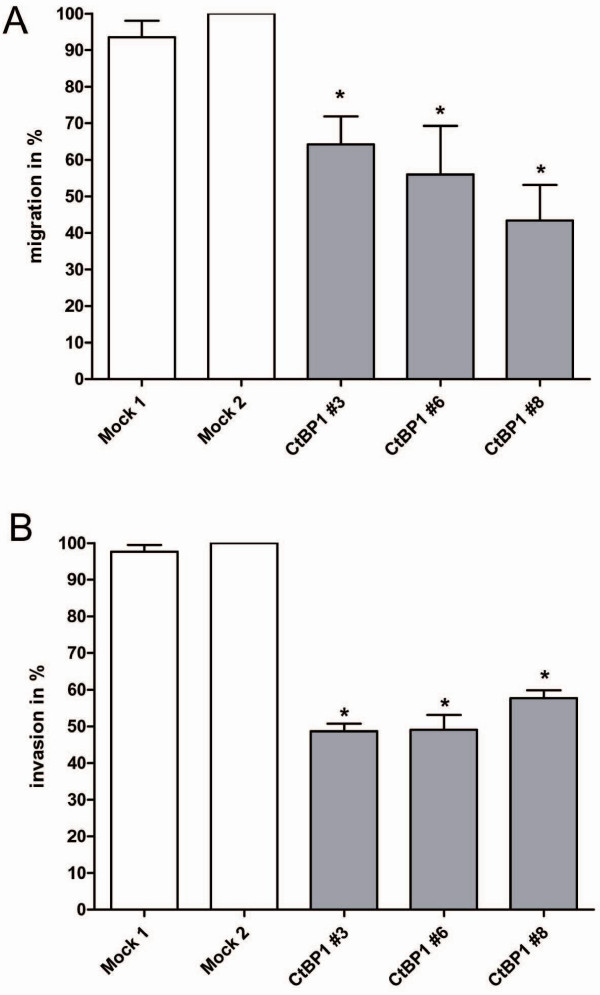
**Migration and invasion in CtBP1 expressing cell clones**. The migratory and invasive potential of the cells was assessed by Boyden Chamber assays using the CtBP1 expressing cell clones. Here, strong reduction in migration (**A**) and invasion (**B**) was observed after CtBP1 re-expression in all three cell clones in comparison to control cells. (*: p < 0.05 compared to Mock 2).

Recently, we showed that CtBP1 expression is lost or strongly reduced in malignant melanoma leading to induction of MIA expression [4]. To find cellular mechanisms regulated by CtBP1 in melanoma we generated CtBP1 expressing melanoma cells by stable transfection to subsequently perform functional assays. The three cell clones selected show strong CtBP1 mRNA and protein expression (Fig. 1A). To confirm the regulation of MIA expression we performed quantitative RT-PCR and revealed a significant reduction of MIA expression in the CtBP1-expressing cell clones compared to mock controls (Fig. 1B). Proliferation and colony formation assays showed no difference in cell growth between CtBP1 expressing and the control clones (data not shown). In contrast, reduction of migration (Fig. 2A) and invasion in Boyden Chamber assays (Fig. 2B) was observed suggesting that CtBP1 and CtBP1-regulated genes control cellular migration.

**Figure 3:**
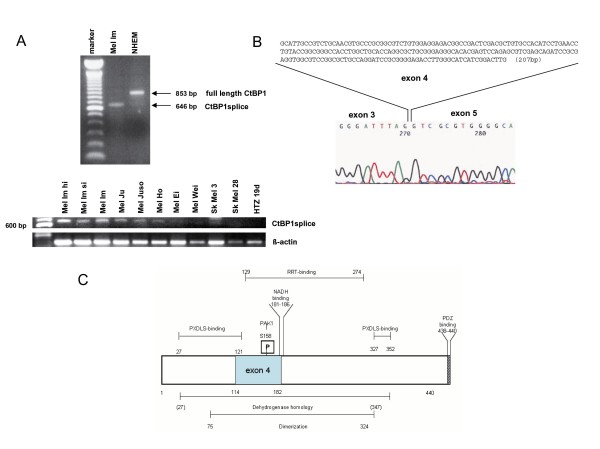
**CtBP1 splice product in melanoma cells**. As observed in Fig. 1A a smaller CtBP1 splice variant is expressed in melanoma cells. (**A**) Using primers flanking the region coded by exon 4 revealed lack of exon 4 in the melanoma cell line Mel Im in comparison to normal human melanocytes (NHEM). The expression of CtBP1splice was shown in eight of eleven melanoma cell lines using PCR (**B**) Sequence analysis of the PCR product shown in (A) confirmed lack of the coding region represented by exon 4. (**C**) In a schematic drawing localization of exon 4 of CtBP1 is displayed.

**Figure 4:**
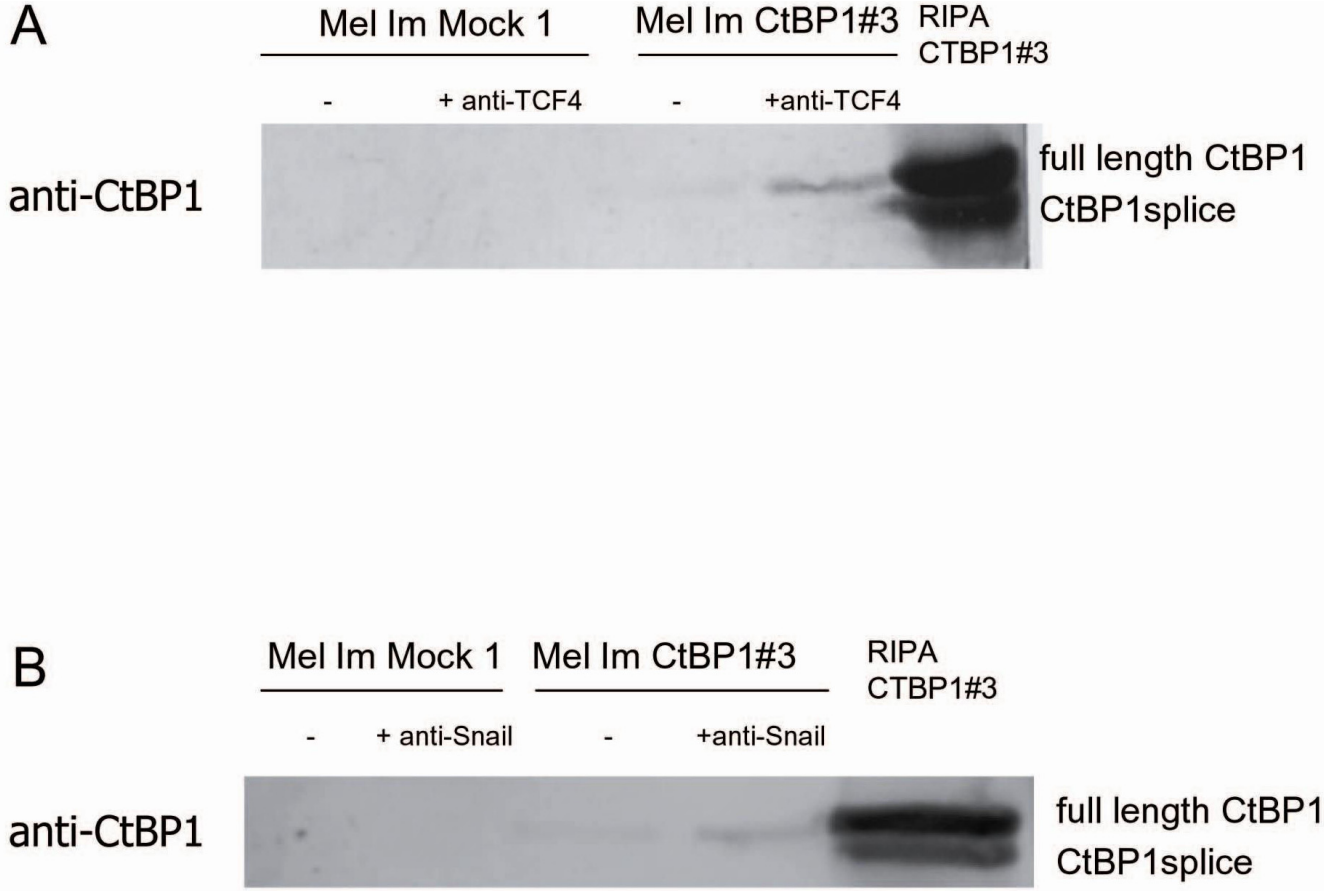
**CtBP1splice does not interact with TCF4 and Snail**. Co-immunoprecipitation were performed using protein lysates of Mel Im Mock 1 control transfected cells and the CtBP1#3 cell clones and were incubated with TCF4-antibody (**A**) or Snail-antibody (**B**). Western blotting with CtBP1-antibody confirmed binding of full length CtBP1 to TCF4 (**A**) and Snail (**B**), respectively, whereas CtBP1splice could not interact with these proteins.

Interestingly, we observed a second minor signal in Western blotting, using the anti-CtBP1 antibody, approximately 5 kDa smaller than full length CtBP1 (RefSeq entry NM_001328) (Fig. 1A). To determine whether this band is due to an alternative splice product of CtBP1 we designed several sets of primer. Using these PCR primers and subsequent sequencing we show that melanoma cells express an alternative CtBP product lacking exon 4 (CtBP1splice) (Fig. 3A, B). Sequence analysis of genomic DNA revealed no mutations in splice sites causing the skipping of exon 4 in melanoma. The expression pattern of CtBP1splice in melanoma is demonstrated in Fig. 3A and revealed the expression of the splice variant in eight of eleven melanoma cell lines. The downregulation of full length CtBP1 in melanoma was previously shown [4]. This results in a protein missing aa 114–182, which is the N-terminal region of the dehydrogenase homology domain and includes a PAK1 phosphorylation site (Fig. 3C). As the nature of this alternative splice product is completely unknown, we sought to determine whether CtBP1 binding to TCF4 (which regulates MIA expression) and Snail is intact. By co-immunoprecipitation we detected TCF4 and Snail binding to full length CtBP1 (in the CtBP1-expressing melanoma cell clones), but none with CtBP1splice (Fig. 4A, B). This suggests that CtBP1splice can not modulate repressor activity on TCF4 and Snail, although other CtBP1 functions may be unaffected.

### CtBP1 target genes

As full length CtBP1 expression is lost in melanoma we hypothesized that CtBP1 acts as a tumor suppressor by negatively regulating genes involved in melanoma development and progression. As CtBP1 is a known repressor of LEF/TCF signaling we first concentrated on known LEF/TCF target genes. MMP7 was found to be down regulated 5-fold by CtBP1 in melanoma, whereas c-myc and laminin gamma 2 were not affected. To define novel putative LEF/TCF target genes in melanoma we screened for genes with LEF/TCF binding sites (TT/GCTTTG) in their promoter using Genomatix software. Genes were chosen showing at least one LEF/TCF binding site as these may be activated if repression by CtBP1 is lost in melanoma and wnt signaling is activated. These genes were analyzed using GEO Profiles (NCBI) in the melanoma array data GDS1989 [11] and we concentrated on genes found to be upregulated during development or progression of the disease. Fourteen of 250 genes analyzed were observed to fit the criteria (LEF/TCF binding site and upregulated in melanoma) applied (table 2). Regulation by CtBP1 of the genes chosen was tested by quantitative RT-PCR in the CtBP1-expressing cell clones compared to the mock control (table 2). Repression of gene expression by CtBP1 was observed in all but two genes (THBS1, MLL5). Col1A2, FAT, SLC26A2 and VCAN were shown to be most strongly regulated. Downregulation of these genes by CtBP1 was confirmed by transient transfection of CtBP1 into the melanoma cell line (Col1A2: 0.31, FAT: 0.52, SLC26A2: 0.72, VCAN: 0.75 compared to mock control set as 1).

**Table 2:** Details on genes regulated by CtBP1 associated to melanoma development Genes with TCF/LEF binding sites in their promoter were analyzed.

Gene symbol	Gene name	TCF/LEF site	**GDS1989 [xfold ind., Nevus set as 1] **[11]			CtBP1 clones versus mock	**Hoek *et al. ***[12]		**x fold ind**.	P <
			**MIS**	**RGP**	**LN met**		**prol**.	**inv**.		

AP1S1	adaptor-related protein complex 1. sigma 1 subunit	+	2.02	2.99	5.23	**0.77**	0.99	1.10	1.11	ns

ATP1B1	ATPase. Na+/K+ transporting. beta 1 polypeptide	+	3.11	3.89	2.63	**0.77**	1.01	2.05	2.03	ns

CLDN11	claudin 11 (oligodendrocyte transmembrane protein)	+	1.06	5.15	2.30	**0.67**	**1.97**	**128.6**	**65.38**	**0.003**

COL1A2	collagen type I alpha 2	+	1.01	2.90	2.00	**0.38**	**6.25**	**34.62**	**5.54**	**0.005**

ENC1	ectodermal-neural cortex (with BTB-like domain)	+	2.58	13.19	14.38	**0.68**	**0.69**	**2.66**	**3.88**	**0.003**

ENPP2	ectonucleotide pyrophosphatase/phosphodiesterase 2 (autotaxin)	+	0.86	1.36	2.31	**0.66**	**1.40**	**4.43**	**3.17**	**0.047**

FAT	FAT tumor suppressor homolog 1 (Drosophila)	+	1.29	2.65	3.29	**0.52**	**0.85**	**1.66**	**1.95**	**0.004**

FSCN1	fascin homolog 1. actin-bundling protein (Strongylocentrotus purpuratus)	+	2.07	4.36	3.21	**0.79**	**0.75**	**2.77**	**3.67**	**0.0002**

FUS	fusion (involved in t(12;16) in malignant liposarcoma)	+	1.70	2.59	3.29	**0.82**	**1.31**	**0.94**	**0.71**	**0.039**

MLL5	myeloid/lymphoid or mixed-lineage leukemia 5 (trithorax homolog. Drosophila)	+	1.77	3.43	3.91	**1.32**	1.22	1.03	0.85	ns

SLC26A2	solute carrier family 26 (sulfate transporter). member 2	+	1.86	4.8	3.11	**0.41**	1.20	1.87	1.56	ns

THBS1	thrombospondin 1	+	1.28	5.91	3.61	**1.2**	**0.27**	**13.59**	**49.87**	**9.46E-06**

TMED4	transmembrane emp24 protein transport domain containing 4	+	0.81	1.75	1.99	**0.74**	**0.91**	**1.73**	**1.89**	**0.002**

VCAN	versican	+	0.88	3.02	4.53	**0.42**	**0.73**	**15.43**	**21.24**	**0.0001**

Using the micro array data GDS1989 [11] upregulation of these genes during melanoma development and progression is shown in columns 4 to 6. The regulation of these genes by CtBP1 was quantified in CtBP1 expressing cell clones by real time-PCR (column 7). For analysis of the proliferative and invasive phenotype the data of Hoek *et al. *[12] were used (column 8, 9). P-value < 0.05 was printed in bold. Abbreviations: MIS, melanoma-in-situ; RGP, radial growth phase; LN met, lymph node metastasis; prol., proliferative phenotype; inv., invasive phenotype.

To get more information about the relevance of the genes found to be regulated by CtBP1 for malignant melanoma we used DNA microarray data obtained from proliferative and invasive phenotype cultures [12]. This showed that expression of 11 CtBP1-regulated genes (73%) were significantly associated with the invasive phenotype recently characterized by Hoek *et al*. [12] and none with the proliferative cluster supporting our functional data that CtBP1-regulated genes control cellular migration (table 2).

## Discussion

CtBP1 is a known co-repressor involved in gene regulation with several other transcription factors. Recently, we described the loss of CtBP1 expression in melanoma [4]. In this study, we aimed to evaluate the importance of this loss and subsequent changes to regulated genes in more detail.

Interestingly, Western blot and RT-PCR studies revealed the existence of a CtBP1 splice variant (CtBP1splice) in melanoma cells. This variant lacks exon 4 (in frame) leading to a CtBP1 variant missing aa 114–182, and does not bind TCF4 or Snail suggesting that CtBP1 co-repressor activity may not be fulfilled by this variant. However, expression of the variant seems to be important in tumors as CtBP1splice was also detected in breast, colon and hepatocellular carcinoma cells which lack full length CtBP1 expression (data not shown). Several other splice forms of CtBP1 have been previously detailed [14], however the splice variant we describe is novel. The lack of the PAK phosphorylation site could lead to changes in the control of nuclear-to-cytosolic translocation and co-repressor function [15]. In addition, NAD binding to CtBP1 and CtBP1 dimerization may be affected. As NAD(H)-dependent dimerization is required for transcriptional repression, repressor functions could also be influenced. Additionally, changes in the two protein binding sites could impact the binding of transcription factors to CtBP1splice [16,17]. CtBP1splice expression after reduction of full length CtBP1 could be important in tumor cells due to general functions recently been described for CtBP as CtBP1 was shown to be involved in Golgi morphogenesis by association to centrosomes [18] and in vesicular trafficking. Interestingly, some publications also hint at a role for CtBP as a tumor promoter [1]. Jin *et al. *describe upregulation of MDR1 by CtBP1 leading to drug resistance [19]. This effect could not be shown to be modulated by histone modification which led the authors to suggest regulation via CtBP1 by an unknown mechanism. In addition, CtBP1 was shown to activate expression of Wnt genes in a TCF-independent manner [20]. It can only be speculated that these tumor promoting effects can be achieved by CtBP1splice. Further studies are necessary to understand the function and role of CtBP1splice in carcinogenesis.

CtBP1 has been speculated to be involved in normal cell growth control. It was shown previously that the binding of CtBP to adenoviral E1A correlates with inhibition of E1A and H-ras cotransformation, tumorigenesis, and metastasis [21]. Furthermore, a repressor function of CtBP on E2F-medited transcription via RB was detected [22]. In our study, proliferation was unchanged after reexpression of CtBP1 whereas migration and invasion assays pointed to a role of CtBP1 in cell migration as reexpression of CtBP1 induced a reduction of the migratory potential. This finding was further supported by detailed analysis of CtBP1-regulated genes. By comparison of genes carrying a LEF/TCF site with cDNA array data (GDS1989) [11] on melanoma fourteen genes showing a correlation with melanoma development and progression were determined. Regulation of these genes by CtBP1 was confirmed performing quantitative RT-PCR. Interestingly, expression of most CtBP1-regulated genes discussed here is associated with the invasive state recently described by Hoek and co-workers [12]. This supports the finding that loss of CtBP1 enhances the migratory behavior of cells.

Only two of the CtBP1-regulated genes were previously described as playing a role in melanoma. ENPP2 (Autotaxin) was originally isolated from melanoma cells and shown to augment the invasive potential of melanoma cells by inducing uPA expression [23,24]. Versican was also previously described to be expressed in melanoma and to be an inducer of metastasis [25]. The remaining genes have not previously been described to play a role in melanoma, however, deregulation and implication in other kinds of cancer has been noted. ATP1B1, an Na_2_K-ATPase, was found to be involved in progression and metastasis of prostate cancer [26]. Collagen type I alpha2 and FUS were found to be deregulated in gastric cancer and speculated to play a role in invasion and metastasis [27]. Changes in Claudin 11 were shown during progression of epithelial tumors leading to acquisition of migratory potential [28]. Upregulation of ENC1, an actin-binding protein, contributes to colorectal carcinogenesis [29]. Regulation of ENC1 gene expression by LEF/TCF was already shown. FAT, a cadherin-family molecule, and FCSN1, a protein involved in the assembly of actin filament bundles, were described to play a role in general cell migratory processes [30,31]. Enhanced FSCN1 expression was associated with increased invasiveness in urinary cancer. TMED4 was significantly induced in invasive adenocarcinoma [32]. In summary, the data available on the functional role of CtBP1-regulated genes hint to an implication in migratory and invasive processes.

It therefore appears that loss of the CtBP1 co-repressor function may be a critical event in the pathogenesis of melanoma. In future, it would be highly interesting to extend the analysis and perform *in vivo *analysis to look at effects of full length CtBP1 upon tumor growth in a nude mouse model.

## Conclusion

In melanoma cells loss of full length CtBP1 expression, a known co-repressor involved in gene regulation with several other transcription factors, is accompanied by induction of a CtBP1 splice variant. This variant lacks exon 4 (in frame) leading to a CtBP1 variant which is unable to bind TCF4 or Snail suggesting that CtBP1 co-repressor activity may not be fulfilled by this variant. Interestingly, in some reports also hint at a role for CtBP as a tumor promoter. The data generated in this study on the functional role of CtBP1-regulated genes suggest an implication in migratory and invasive processes. It, therefore, appears that loss of the CtBP1 co-repressor function may be a critical event in the pathogenesis of melanoma, however, important tumor promoting effects of CtBP can be achieved by CtBP1splice.

## Abbreviations

PXDLS: Pro-X-Asp-Leu-Ser; RRT: Arg-Arg-Thr; aa: amino acids.

## Competing interests

The authors declare that they have no competing interests.

## Authors' contributions

AW carried out most experiments presented in this study, was involved in analysis and interpretation of the data and in drafting of the manuscript. IP first determined the CtBP1 splice form presented in this study, was involved in analysis and interpretation of the data and in drafting of the manuscript. KSH participated in the design of the study and performed the cDNA array analysis. He further was involved in revising the manuscript critically. AKB conceived the study and was responsible for its design and coordination, analysis and interpretation of the data and for drafting of the manuscript. All authors read and approved the final manuscript.

## Pre-publication history

The pre-publication history for this paper can be accessed here:

http://www.biomedcentral.com/1471-2407/9/52/prepub
